# Building a Culture of Health Through the Built Environment: Impact of a Cluster Randomized Trial Remediating Vacant and Abandoned Property on Health Mindsets

**DOI:** 10.1007/s11524-025-00961-4

**Published:** 2025-02-21

**Authors:** Katherine P. Theall, Jasmine Wallace, Amber Tucker, Kimberly Wu, Brigham Walker, Jeanette Gustat, Michelle Kondo, Christopher Morrison, Casius Pealer, Charles C. Branas, Lisa Richardson

**Affiliations:** 1 Celia Scott Weatherhead School of Public Health and Tropical Medicine, Tulane University, 1440 Canal St., Mailstop 8319, New Orleans, LA 70112 USA; 2 Tulane Violence Prevention Institute (VPI), New Orleans, USA; 3 Tulane Mary Amelia Center for Women’s Health Equity Research, New Orleans, USA; 4 US Department of Agriculture (USDA) Forest Service, Northern Research Station, Madison, USA; 5 Columbia University Mailman School of Public Health, New York, NY USA; 6 Columbia Center for Injury Science and Prevention, New York, NY USA; 7 Monash University School of Public Health and Preventive Medicine, Melbourne, Australia; 8 Tulane University School of Architecture, New Orleans, USA; 9 Institute of Women and Ethnic Studies (IWES), Research and Technology Foundation, Inc, New Orleans, USA

**Keywords:** Greening, Vacant property, Cluster randomized trial, Mindsets, Social capital, Neighborhood, Disorder

## Abstract

Changing built environment conditions to impact health mindsets and health equity may be a promising target for public health interventions. The present study was a cluster randomized controlled trial to test the impact of remediating vacant and abandoned properties on factors related to health mindset—including well-being, health interconnectedness, social capital markers, neighborhood disorder, and worry—as well as direct and indirect violence experiences and the moderating role of racial and income segregation on outcomes. A residential cohort of 405 participants from 194 randomly assigned geographic clusters was surveyed over five waves from 2019 to 2023. Compared to clusters with no treatment, participants in clusters where both vacant lots and abandoned homes were treated experienced significant increases in sense of community (83%, 95% CI = 71 to 96%, *p* = 0.01). Among participants in randomization clusters where only vacant lots were treated, there were declines in perceived neighborhood disorder (− 55%, 95% CI = − 79 to − 5, *p* = 0.06) and worry about community violence (− 56%, 95% CI = − 58 to − 12, *p* = 0.06). There was also a moderating effect of racial and income spatial polarization, with the greatest changes in sense of community observed among more deprived areas with both homes and lots treated, and the largest changes in neighborhood worry and disorder were seen in more deprived areas with only lots treated. Remediation of vacant and abandoned properties may be one approach to change some but not all mindsets around health, and the effects may depend on the type of remediation as well as larger neighborhood conditions such as segregation.

## Introduction

Health mindset refers to the collection of beliefs that an individual holds about what factors contribute to their personal health and well-being and their expectations about the efficacy of individual action on influencing health [1, 2]. Holding positive health mindsets and expectations has been linked to many beneficial health outcomes ranging from the implementation of COVID-19 prevention practices [3], to successful post-operative recovery in children [4], to healthy aging in women [5]. While mindsets may shape individual health behaviors, they also can drive community narratives about health and even in health policies and practices and may therefore be key to the adoption of population-level solutions to health and health inequities [1].

Health mindsets are socially and environmentally influenced and can be shaped by several factors, including characteristics of the built environment, through their impact on social capital utilization, collective efficacy, and perceived risk of violence victimization [6]. Indeed, many residents of historically disinvested neighborhoods report low levels of feelings of interconnectedness, poor social integration, and poor psychological well-being [7]. There has been increasing attention on the impact of deteriorated neighborhood conditions such as vacant and abandoned property, given the growing link between deteriorated neighborhood conditions and a range of harmful exposures, for example, to insect vectors, and heavy metal contamination, as well as adverse health outcomes [8, 9]. Vacant lots and abandoned buildings are visible signs of neighborhood disorder and are correlated with violence, fear, and more disorder [10, 11] and may also be linked to health mindset and expectations.

Intentional development of environments in which health is a shared value hinges on the activation of interventions that address mindset and expectations, sense of community, and civic engagement as drivers [12]. High levels of neighborhood disorder may indicate that residents feel disenfranchised, uninvested in their communities, and skeptical that neighborhood beautification and improvement will prove worthwhile, or be of any benefit to them [13]. In many communities that grapple with the environmental, social, and emotional repercussions of property vacancy and neglect, remediating properties could prove an impactful method to support positive health mindsets.

While remediation of vacant and abandoned spaces may be an effective “curve-shifting” (or population-level) approach to health and potentially mindset-changing, effectiveness may be improved with consideration of additional neighborhood forces that shape health. Given the backdrop of larger neighborhood contexts and the important role of racial residential segregation on a variety of health outcomes [14], interventions that attempt to change neighborhoods for health or other outcomes may be more or less efficacious based on levels of segregation. The Index of Concentration at the Extremes (ICE) has previously been used as a proxy of structural racism [15] and used to quantify the uneven distribution of privilege and deprivation within a geographic area—examining not only the disadvantaged but also the advantaged, in contrast to measures such as poverty level.

This study tests the impact of a cluster randomized controlled trial remediating vacant and abandoned property in New Orleans, LA, on well-being and factors related to mindsets and expectations about health. New Orleans has a well-recognized vacant and abandoned property problem and was a prime location to examine the impact of such an intervention on mindsets around health and the moderating role of racial and income segregation.

## Methods

### Study Design

**Fig. 1:**
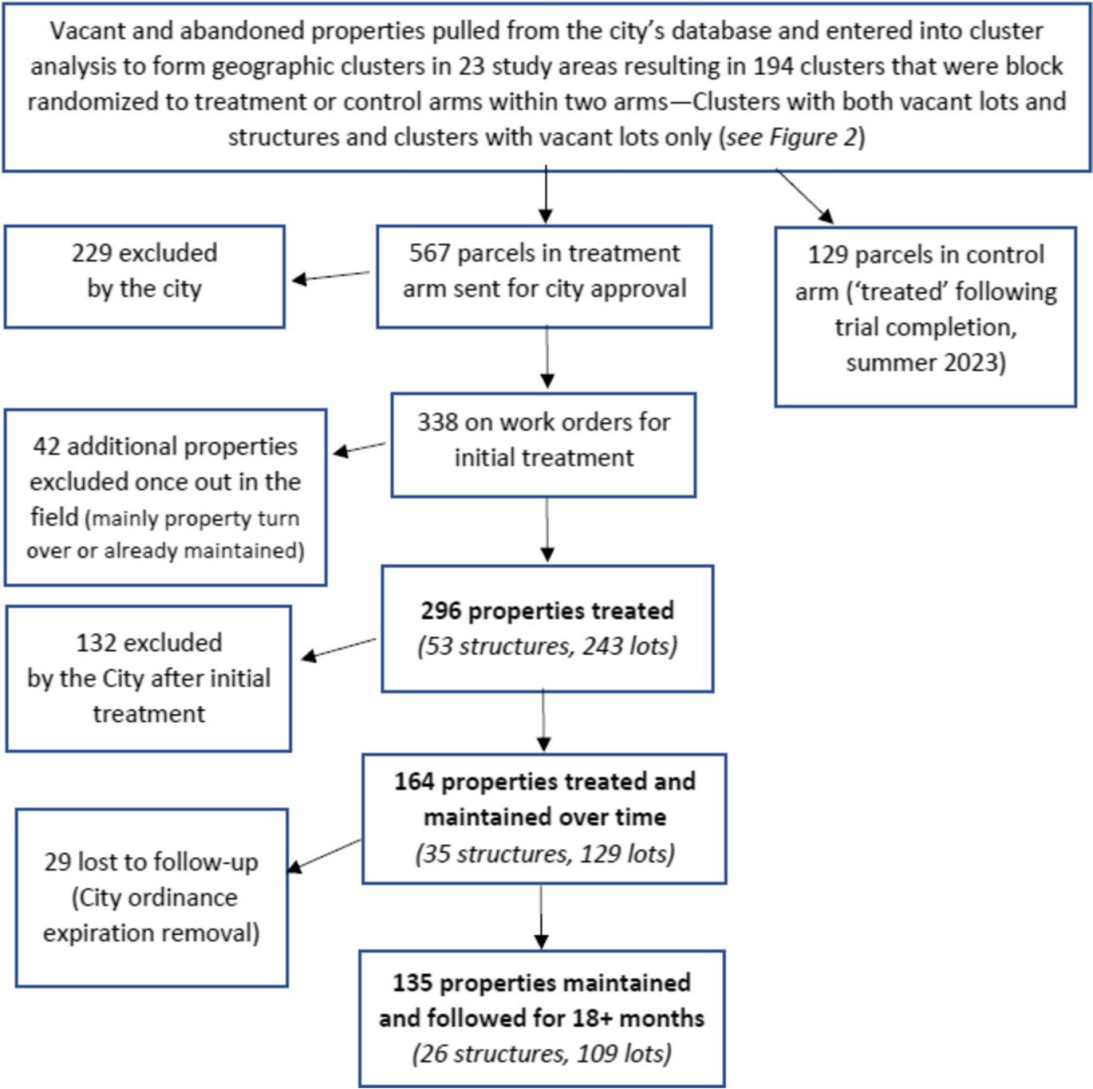
Treatment flow diagram

This analysis was based on data from a cluster randomized controlled trial called the *Healthy Neighborhoods Project* (*HNP*), implemented in 23 of the 73 neighborhoods in New Orleans, to examine the impact of vacant land and building property remediation on violence, health, and mindsets around health. Neighborhoods were selected based on rates of violent crime, which were higher than the city’s mean, and these neighborhoods also had higher rates of poverty than the rest of the city. A list of vacant properties with civil court judgements of code violation under a citywide ordinance was entered into a cluster analysis using STATA to form geographic clusters for randomization, with a roughly 1/8 mile radius, separated enough to prevent contamination. The analysis excluded lots with land area greater than 20,000 square feet and the maximum distance between lots within a cluster was set at 330 feet. It also excluded ineligible clusters with a total land area of ≤ 10,000 square feet and < two lots. From a randomly selected starting point, the program selected eligible clusters whose centroid was within 1320 feet of an included cluster. A total of 400 clusters were formed and those (*N* = 194) in the study neighborhoods were selected for inclusion. As described in Fig. 1, a total of 567 parcels within study clusters were sent to the City for treatment approval, with a total of 296 properties initially treated (53 structures and 243 lots) and 135 maintained over the full study duration (2020 to 2022). Parcels in control clusters (*N* = 129) were also treated beginning in summer 2023.

**Fig. 2:**
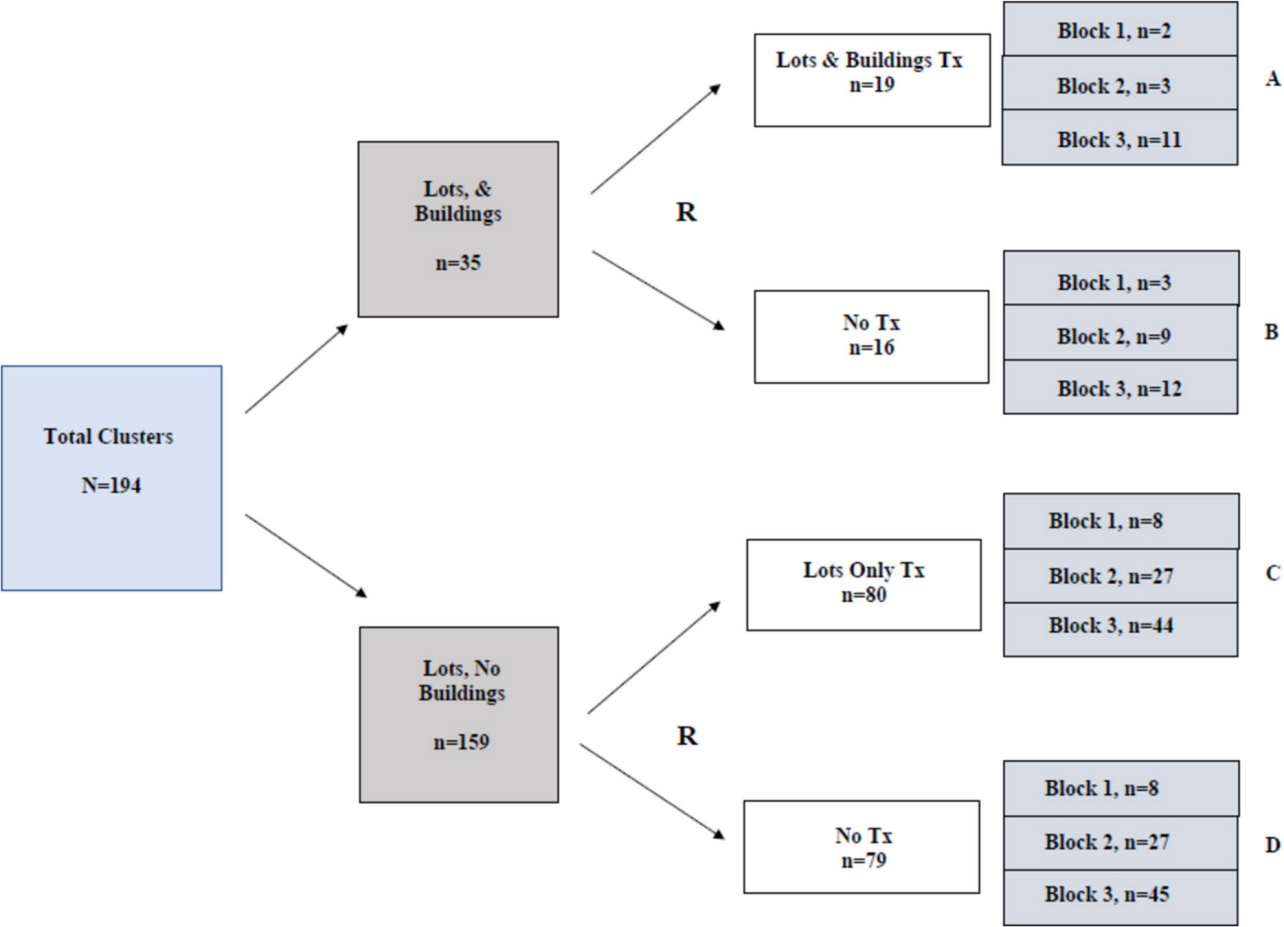
Cluster randomization

**Fig. 3:**
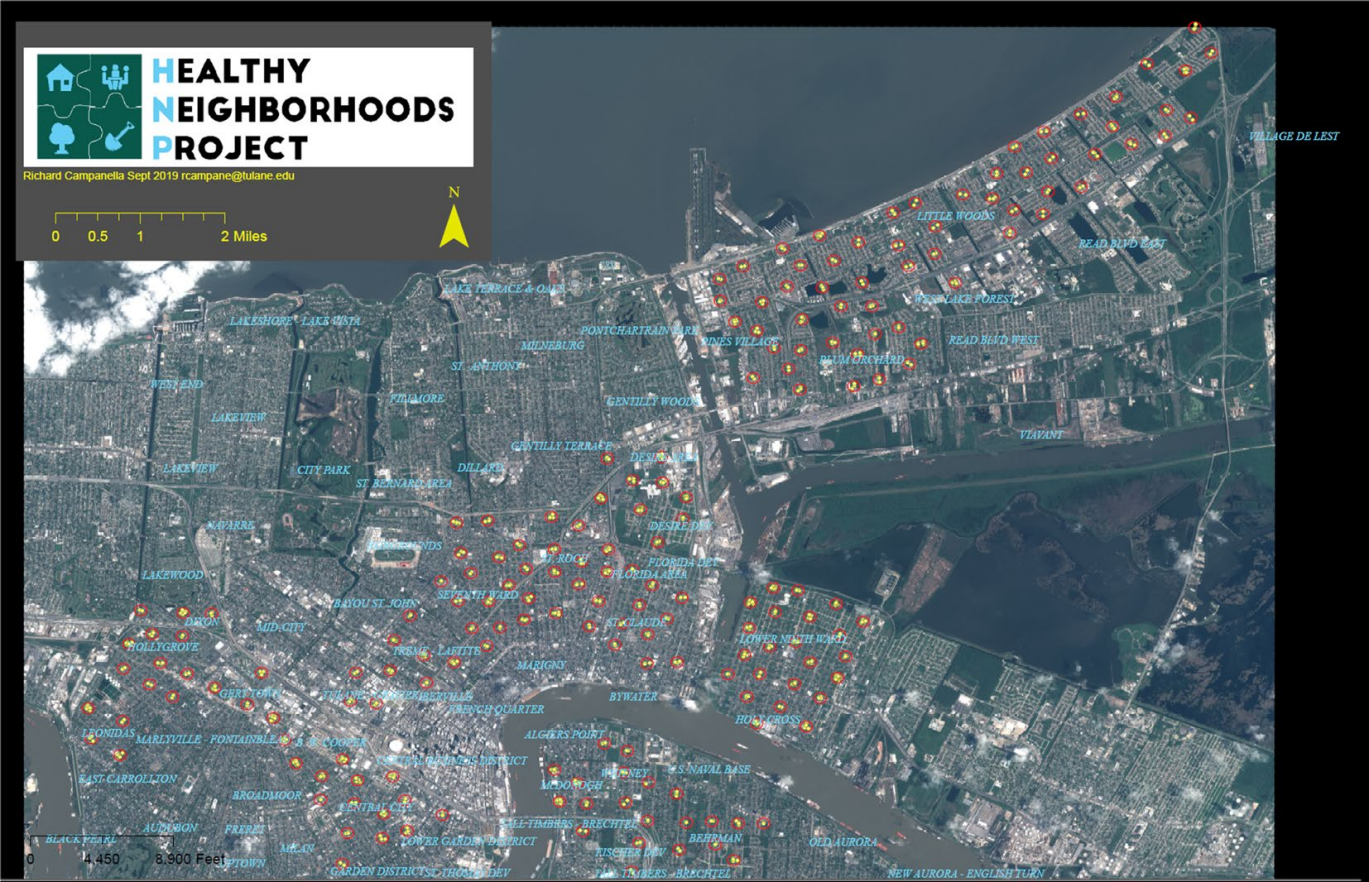
Geographic clusters randomized to treatment or control arms

Cluster randomization of the 194 clusters occurred in two segments as detailed in Fig. 2: clusters with both vacant lots and structures (*N* = 35) and clusters with just vacant lots (*N* = 159). Block random assignment within these groups was performed, with blocks defined as three larger communities based on their separation by main waterways and land use differences. A map of the geographic clusters is shown in Fig. 3.

### Participants and Data Collection

A cohort of 405 participants was surveyed over five waves, beginning in January 2019 and completed in February 2023. Participants were recruited from the 101 intervention and 93 control clusters, and all residents from each cluster were invited to participate. Mailers were sent to valid addresses within each cluster, with approximately 1000 invitations sent out with the study and a Google Voice number for interested residents. There is a 40% response rate and an average of 4 residents per cluster (range, 1 to 5), with 201 residing in treatment and 204 in control clusters—83 in clusters with vacant homes or buildings and 322 in clusters with vacant lots only.

Two surveys were conducted pre-treatment of vacant lots and homes, and three surveys were conducted post-treatment and during the maintenance phase. Surveys were interviewer administered through REDCap™ by trained interviewers. Pre-pandemic, 5% of interviews were conducted in person, and 95% were conducted over the phone. During the pandemic, 100% of interviews were conducted via phone. Wave 2 data collection occurred 4 to 6 months after each participant’s baseline (*n* = 356); Wave 3, or the first post-treatment surveys, approximately 1 to 2 months post-treatment (*n* = 286); Wave 4 surveys 4 to 6 months after Wave 3 (*n* = 263); and Wave 5 conducted 4 to 6 months after Wave 4 (*n* = 223). Post-treatment surveys were staggered by treatment timeline so that respondents in treatment clusters and in control clusters in the same neighborhood received their Wave 3, or first post-treatment survey at a similar time during the calendar year. Approximately 40% of the cohort was lost-to-follow up over the study, which is not surprising given the timing—with COVID-19 starting in March 2020, and Hurricane Ida occurring in August 2021 and leaving much of the city with significant damage and a lack of power for weeks. However, participants were able to complete any of the concurrent waves of survey data collection even if they missed one. Forty-five percent of the baseline sample completed all five surveys. Of the final lost to follow-up, there were 9 deceased, 65 who opted out of continuing (many of whom relocated), and 117 who we were unable to schedule or reach. Given that Wave 2 began shortly after the pandemic and during Hurricane Ida, we suspect that many who we were unable to contact may have moved, and that the number of deceased may be greater. Nonetheless, we observed no differences in those lost to follow-up from those who remained in any of the sociodemographic factors or outcomes of interest, and no differences between treatment and control cluster arms.

### Treatment

**Fig. 4:**
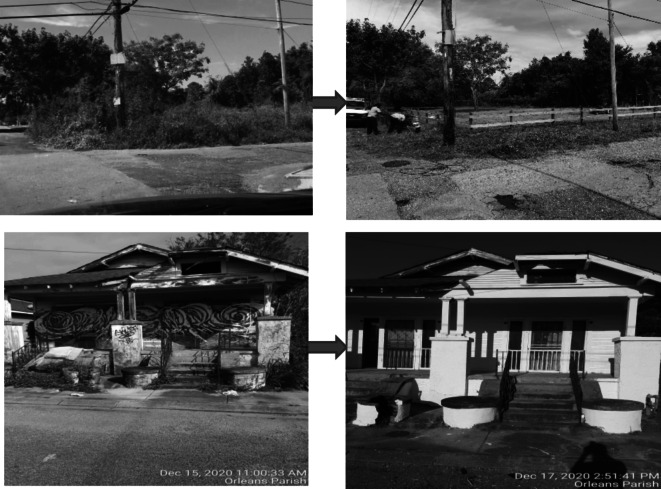
Example of before and after treatment of vacant lots and homes

Implementation began in January 2020 and continued through December 2022. Treatment consisted of both vacant land greening for lots without buildings or homes and abandoned building remediation for lots with buildings or homes. Lot greening consisted of the removal of all refuse, debris, and any overgrowth and the placement of a modest, low post-and-rail wooden fence or bollards around the lot. Building remediation included the removal of any trash or items on or around the structure, removal of broken or boarded windows, and preparation for painting; this was followed by installing new windows and painting where needed. For both lots with and without buildings, a maintenance phase followed treatment and consisted of bi-weekly litter removal, mowing and cleaning during the growing season, and monthly checks and mowing on the off-season for all grass. For buildings, this also included checks and replacement of any windows or paint touch-ups. Examples are shown in Fig. 4.

The Tulane University Institutional Review Board (IRB) approved the study, and the trial is registered with the ISRCTN Registry (#17,742,911).

### Measures

Surveys collected participant demographic information including age, sex at birth, race, marital status, employment status, and education level, as well as a range of measures that assessed mindsets around health, which were the *primary outcomes* of this analysis although not the primary outcomes of the trial itself. Outcomes for the current analysis included well-being, health interconnectedness, social capital markers, neighborhood disorder, and worry—including sense of safety—and direct and indirect violence experiences.

*Well-being* was assessed through a measure made up of five items scored on a five-point scale from 5 = “never” to 1 = “always” with questions related to how the respondent felt over the past month, with statements ranging from “I have felt cheerful and in good spirits” to “my daily life has been filled with things that interest me.” The total score ranged from 5 to 25, with higher scores indicating worse health.

The 4-item *Perceived Stress Scale* [16] was utilized to examine the level of reported stress. Respondents were asked, on a scale from 1 (“always”) to 5 (“never”), and scores were summed across items, ranging from 4 to 20, with higher scores indicating greater perceived stress.

*Health interconnectedness* was assessed through the Health Interdependence measure using three items [17]. One item scored on a 4-point scale from “a lot” = 1 to “not at all” = 4 (“How much would you say that the place where you live affects your own personal health?”), one item on a 3-point scale from “healthy” = 1 to “unhealthy” = 3 (“Overall, would you say that you live in a healthy community, an unhealthy one, or one that is somewhere in between?”), and the last item on overall health scored on a 4-point scale from “excellent” = 1 to “poor” = 4 (“Would you say your health in general was…”). Items were recoded into binary variables representing the belief that place impacts health (“a lot” or “some” vs. “not much” or “not at all”), that the respondent believed their neighborhood is a healthy one (vs. “unhealthy” or “somewhere in between”), and an excellent or good perception of their own health (vs. “fair” or “poor”).

*Social capital* was assessed with three separate indicators—*sense of community*, *collective efficacy*, and *civic engagement*. Sense of community was measured using the sense of community index (SCI), a validated tool that measures perceptions of connection and membership to a group or community [17]. The SCI includes 12 items, scored on a “Mostly true/Mostly false” scale for each item, resulting in total scores ranging from 0 to 12. The SCI was designed to include an overall score and four subscale scores that capture more specific constructs for sense of membership, sense of influence, reinforcement of needs, or shared emotional connection. SCI demonstrated strong reliability (Cronbach’s alpha = 0.85).

Participants’ perceptions of neighborhood collective efficacy were assessed with survey items taken from measures developed by Morenoff and colleagues [18] which included 8 items scored on a 5-point scale ranging from 1 (“strongly disagree”) to 5 (“strongly agree”), for total scores ranging from 8 to 40. Lower scores indicated a lower sense of collective efficacy. The measure demonstrated strong internal consistency in our sample (Cronbach’s alpha = 0.85).

Civic engagement was assessed with “yes” or “no” responses related to questions about how individual civic engagement might influence governmental decisions around health issues [17], combined into a summary score ranging from 0 to 6. Participants indicated whether they: “voted for or against a candidate for public office because of their position on a health problem or issue”; “contributed time or money to an organization working to prevent or cure a specific disease like cancer or HIV/AIDS”; “contributed time or money to an organization working to make the community a healthier place to live”; “contributed time or money to an organization working to pass a government health law or policy”; “volunteered for a group/board/committee/council that addresses health-related issues and activities for my community”; “written an email, letter or signed a petition on some health problem or issue.”

*Neighborhood disorder and worry* were measured with 17 binary (“yes” = 1/ “no” = 0) items indicating whether the respondent is worried about: drug dealers or users hanging around; having property stolen; walking alone during the day; walk alone at night; letting children go outside during the day; letting children go outside during the night; being robbed; being murdered; litter or trash on the sidewalks or streets; graffiti on buildings and walls; abandoned cars; vacant, abandoned, or boarded up buildings; houses and yards not kept up; drunks hanging around; gang activity; different social groups who do not get along with each other; and gun violence. Items were combined into an overall neighborhood disorder and worry score, ranging from 0 to 10 (Cronbach’s alpha = 0.92). Items were also broken down further to represent sub-scales for *neighborhood disorder*, *worry about safety or fear of being victimized*, and *worry about community violence*.

In addition to the items above, *sense of safety* was also measured with a perceived safety scale, measured on a scale from 1 to 10 (where 1 means “completely safe” and 10 means “extremely dangerous”) and with the question of how safe participants felt to walk around alone in their neighborhood after dark in the last month.

*Direct and indirect violence experiences* were measured with a series of six binary items (“yes” = 1 and “no” = 0) that asked respondents whether the following occurred in the last month: involvement in any fights in your neighborhood; seen someone shoved, kicked, or punched in your neighborhood; hearing gunshots in your neighborhood; carrying a gun in your neighborhood; experienced any kind of physical violence by friends or family members; and experienced any kind of physical violence by strangers. The variables were also combined into a summative score that ranged from 0 to 6, with higher values indicating greater exposure.

The *primary exposure* was measured at the cluster level as a binary variable (treatment vs. control cluster). A secondary exposure captured the level of treatment (control, moderate level, high level), which included information on the frequency of treatment maintenance (number of times treated and average time between treatments), whether any parcels in the cluster included fences installed, whether any dumping was removed on the parcel, the average cluster land size treated, and the number of lots treated within each cluster. Treatment clusters at the 75th percentile or higher on these factors were considered a higher level of treatment, while others were considered moderate. Fifty-six percent of the 194 clusters were in the treatment group, with 52% considered moderate and 48% high levels of treatment.

The primary cluster level moderator examined was ICE [15], estimated for every census tract in New Orleans, LA, using 2014–2018 American Community Survey (ACS) 5-year estimates of household income by race/ethnicity by taking the difference between the number of Non-Hispanic (NH) white persons whose annual household income was greater than or equal to the 80th percentile (> $100,000) minus the number of NH Black persons whose household income was less than the 20th income percentile (< $25,000), divided by the total population with known income in the tract. Values ranged from − 1 (indicating 100% of the population is concentrated in the most deprived group) to 1 (indicating 100% of the population is concentrated in the most privileged group), and for the purpose of testing effect modification, ICE was categorized as high or low based on the median value of the cluster sample.

### Statistical Analysis

Multilevel repeated measures regression was employed, utilizing generalized estimating equations (GEE), to examine the impact of treatment over time, with participants nested within clusters and accounting for repeated measures over time among participants. Regression models were analyzed separately for each outcome. Moderation effects were tested with two-way interaction terms in the regression model and also stratified by ICE [19]. For ease of interpretation, we converted coefficients from models into the percentage change in outcomes. All analyses were conducted with SAS version 9.4.

## Results

Characteristics of participants are presented in Table 1. Approximately 70% of respondents self-identified as women, 76% as Black, 3% as Hispanic or Latine, and 48% of participants were employed at least part-time. Participants in all clusters reported moderate levels of overall neighborhood disorder and worry. This trend was the same for the three domains of fear of being victimized or involved in violence, neighborhood disorder, and neighborhood violence. Participants in control clusters reported slightly greater neighborhood disorder than participants in treatment clusters at baseline, although the trend was only marginally significant (*p* < 0.10). Residents in both cluster groups reported moderate levels of perceived safety.

**Table 1:** Characteristics of residential cohort members by treatment level of cluster

	Total ***N*** = 405 % or mean (± s.d.)	Resides in treatment cluster with structure and lots ***N*** = 42 % or mean (± s.d.)	Resides in treatment cluster with lots ***N*** = 160 % or mean (± s.d.)	Resides in control cluster ***N*** = 203 % or mean (± s.d.)
**Sex**				
Female	69.55%	63.41%	71.70%	68.63%
Male	30.45%	35.59%	28.30%	31.37%
** Age** (years)	51.71 (16.04)	55.41 (14.66)	54.28 (15.38)	49.03 (16.39)
**Race**				
American Indian or Alaska Native	-	-	-	-
Asian	0.25%	-	0.63%	-
Native Hawaiian or Pacific Islander	0.25%	-	-	0.49%
Black	75.25%	88.10%	73.13%	76.96%
White	16.09%	9.52%	20.00%	15.20%
Other or multiple	6.19%	2.38%	6.25%	7.35%
** Hispanic or Latino ethnicity** (yes)	3.23%	4.88%	2.53%	-
Education*				
High school graduate/GED or less	41.03%	51.22%	39.35%	39.80%
Some college, 2-year junior/community college/vocational/business/trade school	30.51%	31.71%	25.81%	34.18%
4-year college/university graduate or more	28.46%	17.08%	34.84%	26.02%
**Employment**				
Working full time, 35 h/week or more	35.91%	31.71%	34.81%	37.75%
Working part time, less than 35 h/week	13.22%	12.20%	10.76%	15.20%
Unemployed	27.43%	34.15%	22.78%	22.55%
Other	23.44%	21.95%	31.65%	24.51%
**Relationship status**				
Married, or living with partner	25.43%	14.29%	30.00%	24.39%
Divorced/separated or widowed	22.71%	38.10%	20.63%	21.47%
Single	39.75%	35.71%	39.38%	40.49%
More than one of the above	12.10%	11.90%	10.00%	13.66%
** Number of neighbors known**	5.24 (5.08)	5.54 (6.23)	5.53 (4.90)	4.94 (4.96)
** Neighborhood disorder and worry** (range = 1 to 10)	4.47 (2.13)	4.04 (2.11)	4.35 (2.23)	4.65 (2.04)
Fear of being victimized or involved in violence	5.11 (2.39)	4.49 (2.42)	5.04 (2.48)	5.28 (2.30)
Neighborhood disorder	4.18 (2.57)	3.91 (2.79)	3.94 (2.56)	4.47 (2.55)
Neighborhood violence	3.67 (2.62)	3.28 (2.44)	3.57 (2.62)	3.83 (2.65)
** Perceived safety** (range = 0 to 10)	5.52 (3.16)	4.93 (3.24)	5.52 (3.16)	5.65 (3.16)
** Sense of community score** (range = 0 to 3)	2.36 (0.72)	2.32 (0.76)	2.41 (0.72)	2.33 (0.72)
Membership	2.60 (0.77)	2.66 (0.79)	2.66 (0.74)	2.54 (0.78)
Influence	2.11 (0.99)	2.03 (1.05)	2.15 (1.03)	2.09 (0.96)
Reinforcement	2.43 (0.92)	2.35 (0.92)	2.47 (0.91)	2.41 (0.94)
Shared connection	2.34 (0.96)	2.38 (0.94)	2.37 (0.95)	2.31 (0.97)
** Collective efficacy** (range = 1 to 5)	3.46 (0.73)	3.39 (0.77)	3.52 (0.75)	3.42 (0.71)
Social control	3.77 (0.82)	3.64 (0.76)	3.81 (0.85)	3.76 (0.82)
Social cohesion	3.15 (0.81)	3.15 (0.95)	3.23 (0.82)	3.09 (0.77)
** Total civic engagement score** (range = 0 to 2)	2.20 (1.74)	1.71 (0.25)	1.62 (0.30)	2.26 (1.75)
**Health interdependence**				
Feel like residential place affects personal health some or a lot*	56.97%	41.46%	61.64%	56.44%
Perceived health of community as healthy†	33.00%	42.86%	37.74%	27.23%
** Perceived health status as good or excellent**	69.90%	69.05%	71.70%	68.81%
** Perceived well-being** (range = 5 to 25)	19.45 (3.91)	20.31 (3.92)	19.41 (4.10)	19.26 (3.77)
** erceived stress scale** (range = 4 to 20)	8.41 (3.12)	7/98 (3.13)	8.62 (3.18)	8.36 (3.08)

Note. Percentages and estimates based on non-missing data (< 5% were missing across all variables)

^†^*p*-value < 0.05; *p-value < 0.10

Participants in both treatment and control clusters also indicated high to moderate baseline levels of sense of community and collective efficacy, but lower levels of civic engagement. With respect to health interdependence, more than half of the sample (56.97%) of participants believed that the residential place affects personal health some or a lot, yet only one-third felt their community was healthy. Approximately 70% of participants reported that their health status was good or excellent alongside high scores of perceived well-being and low to moderate levels of perceived stress.

There were few differences between residents in treatment versus control clusters, as shown in Table 1, with the exception of education level which was slightly higher for residents in treatment clusters with lots only, followed by those in the control clusters, and also in perceptions of health interdependence, with a greater proportion of those in the treatment clusters with lots only feeling that place has a great effect on personal health and a greater proportion of those in treatment clusters with both lots and structures having a better perception of their neighborhood’s health.

The impact of treatment is shown in Table 2. For participants in clusters with both vacant lots and homes at baseline, in crude models (Model 1, column 1), we observed significant increases in sense of community overall (83%, 95% CI = 71 to 96%, *p* = 0.01), as well as subscales of reinforcement (73%, 95% CI = 51 to 94%, *p* = 0.01) and shared emotional connection (77%, 95% CI = 57 to 97%, *p* = 0.03). Participants in treatment clusters of this randomization arm also reported increases in levels of collective efficacy, although the trend was marginally significant (73%, 95% CI = 51 to 94%, *p* = 0.09). We also observed a decrease in fear of victimization among participants in treatment versus control clusters in this randomization arm, although also marginal (− 84%, 95% CI = − 88 to − 81%, *p* = 0.08).

**Table 2:** Impact of treatment on mindsets around health by randomization group

	Clusters with vacant lots and structures vs. control (# observations = 374)		Clusters with vacant lots only vs. control (# observations = 1441)	
	% Change (95% CI)	*p*-value	% Change (95% CI)	*p*-value
Perceived well-being	33 (− 31 to100)	0.23	79 (− 12 to 88)	0.54
Perceived stress scale	− 41 (− 56 to 62)	0.17	− 88 (− 24 to 54)	0.65
Health interconnectedness				
Neighborhood impacts health	− 84 (− 44 to 58)	0.58	− 15 (− 58 to 16)	0.31
Neighborhood is a healthy one	2 (− 52 to 99)	0.96	15 (− 23 to 62)	0.43
Excellent or good health perception	82 (− 45 to 60)	0.56	1 (− 47 to 44)	0.94
Sense of community (SOC) overall	**83 (71 to 96)**	**0.01**	99 (− 92 to 93)	0.69
SOC membership	89 (− 2 to 92)	0.20	93 (84 to 98)	0.10
SOC influence	85 (− 7 to 93)	0.19	89 (− 88 to 99)	0.54
SOC reinforcement	**73 (51 to 94)**	**0.01**	93 (82 to 96)	0.20
SOC shared emotional connection	**77 (57 to 97)**	**0.03**	91 (− 85 to 97)	0.90
Collective efficacy	**89 (77 to 98)**	**0.09**	93 (81 to 95)	0.26
Civic engagement	85 (54 to 84)	0.30	86 (79 to 99)	0.58
Neighborhood worry and disorder				
Overall	− 41 (− 76 to − 33)	0.16	− 66 (− 26 to 6)	0.11
Perceived neighborhood disorder	− 58 (− 68 to − 35)	0.38	** − 53 (− 79 to − 5)**	**0.06**
Fear of victimization	− 77 (− 33 to 78)	0.62	** − 84 (− 88 to − 81)**	**0.08**
Worry about community violence	− 70 (− 31 to 28)	0.56	** − 56 (− 58 to − 12)**	**0.06**
Perceived safety	− 48 (− 44 to 56)	0.29	− 85 (− 36 to 67)	0.54
Direct and indirect violence experiences	− 6 (− 68 to 80)	0.43	− 89 (− 77 to 10)	0.13

Note. Models included block, wave, and baseline value of outcome and based on non-missing data (< 10% of outcomes for overall observations over time)

Data in bold indicate significant or marginally significant results

For participants in randomization clusters with vacant lots only (Table 2, column 2), in crude models, we observed declines in perceived neighborhood disorder (− 53%, 95% CI = − 79 to − 5%, *p* = 0.06) and worry about community violence (− 56%, 95% CI = − 58 to − 12%, *p* = 0.06), although both only marginally significant. Utilizing varying levels of treatment (high, moderate, control) versus treatment vs. control revealed similar changes, albeit greater in magnitude for clusters with higher levels of treatment.

Table 3 presents the results of effect modification by ICE, with Model 1 depicting the impact of the intervention by cluster randomization group for clusters within higher ICE neighborhoods (i.e., neighborhoods with greater privilege and less spatial racial and economic residential segregation) and Model 2, the impact among participants in clusters within lower ICE neighborhoods (i.e., neighborhoods with greater disadvantage and more spatial racial and economic residential segregation). All models were adjusted for block, wave of data collection, and baseline value of the outcome. Among participants in randomization clusters with both vacant structures and lots and within high ICE neighborhoods, we observed significant increases in sense of community and, specifically, the sense of influence (30%, 95% CI = 4 to 67%, *p* = 0.08) and sense of reinforcement that community can provide (68%, 95% CI = 13 to 86%, *p* = 0.001), albeit marginal for sense of influence. Among participants in randomization clusters with only vacant lots (Model 1, Column 2, Table 3) and within high ICE neighborhoods, we observed no significant changes in the outcomes of interest. Utilizing varying levels of treatment (high, moderate, control) versus treatment vs. control revealed similar changes, albeit greater in magnitude for clusters with higher levels of treatment.

**Table 3:** Effect modification by index of Concentration at the Extremes (ICE)

	Clusters with vacant lots and structures vs. control (# observations = 115)			Clusters with vacant lots only vs. control (# observations = 527)	
	% Change (95% CI)		*p*-value	% Change (95% CI)	*p*-value
Model 1. High ICE clusters (greater privilege)*					
Perceived well-being	49 (− 30 to 90)		0.93	71 (− 28 to 89)	0.85
Perceived stress scale	− 46 (− 15 to 62)		0.53	− 85 (− 93 to 63)	0.15
Health interconnectedness					
Neighborhood impacts health	21 (− 10 to 49)		0.77	− 5 (− 44 to 36)	0.82
Neighborhood is a healthy one	1 (− 23 to 21)		0.98	26 (− 3 to 91)	0.41
Excellent or good health perception	22 (− 22 to 35)		0.33	42 (− 32 to 83)	0.21
Sense of community (SOC) overall	81 (57 to 95)		0.12	91 (− 62 to 99)	0.32
SOC membership	77 (71 to 98)		0.84	81 (− 96 to 96)	0.93
SOC influence	**30 (4 to 67)**		**0.08**	80 (− 84 to 97)	0.56
SOC reinforcement	**68 (13 to 86)**		**0.001**	87 (− 48 to 99)	0.93
SOC shared emotional connection	83 (45 to 93)		0.20	88 (− 55 to 91)	0.56
Collective efficacy	86 (− 45 to 98)		0.36	93 (84 to 96)	0.12
Civic engagement	16 (− 74 to 97)		0.60	91 (− 59 to 98)	0.74
Neighborhood worry and disorder					
Overall	− 84 (− 16 to 48)		0.96	− 84 (− 22 to 52)	0.62
Perceived neighborhood disorder	− 83 (− 42 to 98)		0.66	− 66 (− 7 to 61)	0.36
Fear of victimization	− 29 (− 18 to 24)		0.34	− 72 (− 38 to − 93)	0.12
Worry about community violence	− 72 (− 5 to 60)		0.42	− 95 (− 56 to 66)	0.81
Perceived safety	− 18 (− 98 to − 35)		0.18	− 51 (− 80 to 23)	0.15
Direct and indirect violence experiences	− 1 (− 46 to 48)		0.98	− 56 (− 95 to 65)	0.51
Model 2. Lower ICE clusters (more deprivation)* (# observations = 203) (# observations = 507)					
Perceived well-being		69 (− 34 to 29)	0.57	21 (− 58 to 82)	0.55
Perceived stress scale		− 29 (− 97 to 42)	0.28	− 30 (− 63 to 59)	0.27
Health interconnectedness					
Neighborhood impacts health		21 (− 24 to 59)	0.62	72 (1 to 91)	0.24
Neighborhood is a healthy one		2 (− 18 to 10)	0.96	8 (− 47 to 73)	0.73
Excellent or good health perception		**24 (− 3 to 47)**	**0.06**	− 25 (− 44 to 80)	0.89
Sense of community (SOC) overall		**83 (68 to 99)**	**0.04**	87 (− 12 to 99)	0.98
SOC membership		**75 (56 to 96)**	**0.02**	**76 (65 to 89)**	**0.05**
SOC influence		**70 (− 1 to 99)**	**0.05**	77 (− 80 to 84)	0.82
SOC reinforcement		**68 (45 to 92)**	**0.001**	89 (73 to 95)	0.18
SOC shared emotional connection		58 (− 9 to 84)	0.20	84 (− 81 to 98)	0.87
Collective efficacy		**86 (72 to 99)**	**0.06**	71 (− 7 to 89)	0.24
Civic engagement		66 (24 to 91)	0.11	74 (− 64 to 95)	0.74
Neighborhood worry and disorder					
Overall		− 87 (− 29 to 57)	0.63	** − 45 (− 47 to − 12)**	**0.05**
Perceived neighborhood disorder		− 34 (− 88 to 44))	0.29	** − 33 (− 36 to − 31)**	**0.07**
Fear of victimization		− 69 (− 63 to 10)	0.37	** − 37 (− 40 to − 28)**	**0.06**
Worry about community violence		− 44 (− 88 to 23)	0.40	− 55 (− 70 to 6)	0.15
Perceived safety		− 55 (− 69 to 20)	0.48	** − 25 (− 48 to − 3)**	**0.04**
Direct and indirect violence experiences		− 70 (− 89 to 90)	0.30	** − 43 (− 99 to − 14)**	**0.05**

^*^All models adjusted for block, wave, and baseline value of outcome based on non-missing data (< 10% of outcomes for overall observations over time). *ICE* Index of Concentration at the Extremes, where ≥ mean ICE was classified as high ICE

Data in bold indicate significant or marginally significant results

Among participants in randomization clusters with both vacant structures and lots and within lower ICE neighborhoods, we observed significant increases in sense of community overall (83%, 95% CI = 68 to 99%, *p* = 0.04) as well as increases in community membership (75%, 95% CI = 56 to 96%, *p* = 0.02), sense of reinforcement that community can provide (68%, 95% CI = 45 to 92%, *p* = 0.001), and influence (70%, 95% CI = − 1 to 99%, *p* = 0.05), albeit marginal for the later. We also observed marginally significant increases in perceived health (24%, 95% CI = − 3 to 47%, *p* = 0.06) and collective efficacy (86%, 95% CI = 72 to 99%, *p* = 0.06). Among participants in randomization clusters with only vacant lots (Model 2, Column 2, Table 3) and within lower ICE neighborhoods, we observed marginally significant increases in community membership (76%, 95% CI = 65 to 89%, *p* = 0.05), significant decreases in perceived unsafety (− 25%, 95% CI = − 48 to − 3%, *p* = 0.04), and marginally significant decreases in overall neighborhood disorder and worry (− 45%, 95% CI = − 47 to − 12%, *p* = 0.05), perception of neighborhood disorder (− 33%, 95% CI = − 36 to − 31%, *p* = 0.07), fear of victimization (− 37%, 95% CI = − 40 to − 28%, *p* = 0.06), and direct and indirect violence experiences (− 43%, 95% CI = − 99 to − 14%, *p* = 0.05).

## Discussion

This study examined the impact of a cluster randomized controlled trial remediating vacant and abandoned property on factors related to mindsets and expectations about health and the moderating role of racial and income segregation on outcomes. We found that remediating vacant and abandoned properties had an impact on select factors related to mindsets and expectations about health, but that it varied both by the level of property remediation and the level of racial and income segregation in neighborhoods. Differences by level of property remediation were expected to some extent, given the nature of the vacancy and the fact that cleaning and greening vacant lots may be more likely to increase public interaction by providing neutral or utilitarian spaces for residents [20], whereas vacant homes and buildings are typically inaccessible.

With respect to health-related outcomes such as perceived well-being, stress, health, and health interconnectedness, we observed no overall effect across intervention arms. However, we did find increases in perceived good or excellent health among respondents in treatment clusters in less privileged areas where both vacant lots and homes were treated. Given that previous observational studies have shown decreases in stress among residents living in areas exposed to vacant lot greening compared to controls [21], we hypothesized that treatment would impact levels of stress or well-being, but this was not the case in this study. However, given the timeframe during which the trial took place—during the height of the COVID pandemic—levels of stress and lower well-being may have been greatly impacted by more than just local neighborhood changes. While we hypothesized that the treatment may also impact the level of health interconnectedness residents felt for their residential neighborhood, we did not find differences between those in treatment and control clusters, which also may have been impacted by COVID.

In terms of social capital indicators, we observed significant increases in sense of community, though only among participants in clusters where both vacant lots and homes were treated. The greatest impact was observed across several subscales of sense of community among participants in these clusters located in areas with greater deprivation. To the best of our knowledge, no intervention trials have examined specifically the impact of vacant property remediation on validated measures of social capital, but given that vacant properties also reduce community cohesion [22], our observed finding confirms our hypothesis. Other work examining community-engaged lot repurposing and beautification [23, 24] has observed improved social interaction with neighborhood residents and sense of community. Some of greening and remediation programs that have involved the community have also shown more connection to neighborhoods after participating [25]. While the impact of vacant building remediation on neighborhood markers of social capital and sense of community may be greater in community-engaged or led “bottom-up” programs, results suggest that there still may be an impact of “top-down” population-level programming, at least in areas where both vacant lots and homes were treated and in areas with greater deprivation to begin with. This may be due to simple “cues to care” whereby residents become more engaged and look out for each other [26], the spatial contagion of greening and lot remediation (or the “greening hypothesis”) [27], and Busy Streets Theory [28] which highlights safe streets where businesses are flourishing, homes are occupied and well maintained, and residents are socially engaged with one another.

With respect to neighborhood disorder and worry, decreases in perceived disorder, fear of victimization, and worry about community violence were seen among participants in clusters where only vacant lots were treated. This remained when examining across levels of racial and income segregation, although only seen among participants in the more deprived areas based on segregation and with significant decreases in worry overall, as well as in perceived feeling of being unsafe and in experiences of violence. These findings also corroborate previous studies to some extent, with previous experimental studies of vacant lot greening observing increases in perceived safety for those living near greened lots versus controls [6, 29, 30]. This may be due to actual decreases in violence as the majority (71%) of intervention studies on greening interventions have observed lowered crime, including gun violence [9]. However, the impact of vacant property remediation may depend on the type of treatment, as a trial testing the impact of remediation of vacant homes found no impact on perceived safety among residents [31], somewhat similar to our finding in that the effect on perceived violence and disorder was observed primarily among the vacant lot treatment clusters specifically.

Across several outcomes, greater impacts were observed among areas with higher levels of racial and income segregation or deprivation, similar to many greening studies which have found that the effect may be concentrated in lower socioeconomic areas [9, 32]. Improvements to the quality of vacant homes and lots and other types of greenspaces in these settings may aid in changing some of the pervasive health inequities observed by neighborhoods. While they do not address the policies and practices that have shaped and continue to shape many neighborhoods, they do offer one potential, lower cost means of changing basic drivers of poor health, specifically in the built environment. Interventions that change the neighborhood environment may also create awareness of the inequities that exist across communities, particularly for racial and ethnic minoritized groups. Such awareness-building can set the stage for changing mindsets and larger mobilization and action to build a culture of health and reduce inequities.

However, such an approach is not without its challenges, including public versus private ownership of land which may affect the speed with which remediation can be undertaken. In this study, we were able to work directly with the City’s Code Enforcement Division and under a city-wide ordinance that allowed the City and partners to treat the properties of unresponsive owners who were in violation of city ordinances. While we reached out offering free remediation to private property owners, we heard back from less than 20%. City policies may be helpful in this case and initiatives for vacant lot buy-out or entrance of vacant land into a public trust have had some successes.

This study is not without limitations, including a sample from relatively low-income and high-violence areas in a Southern US city, limiting generalizability. Our data also represents self-reported survey responses, subject to information and social desirability biases, although this would be the case in both the treatment and control groups, and there is little reason to suspect that it would uniquely bias the results. Additionally, the number of residents per cluster was small for some clusters; however, this may not have been a significant issue given the number of groups [33]. Furthermore, there is the possibility that COVID further impacted results given that many were at home more often or may have spent more time outside during the pandemic. We would expect effects to be potentially greater during COVID if that was the case; however, most of the clusters were in lower-income communities that may have also included a greater proportion of essential workers, and, therefore, travel or outdoor time may not have changed substantially. As discussed above, COVID certainly impacted our lost-to-follow-up, but this did not appear to differ by cluster. Finally, given the size and scope of the trial, community engagement across all 23 neighborhoods was a challenge. While community engagement is a critical component in community greening initiatives [25], we were limited in the level of community engagement that could be achieved in this study and also by the study design as we did not want to contaminate treatment or bias findings. Given that, we had only broader city-wide engagement during the actual trial and not within the specific neighborhoods of study cluster locations. For recruitment, residents were given general information about a study aiming to understand the impact of neighborhood on health and well-being, and nothing about the intervention itself.

In summary, remediation of vacant and abandoned property may be one approach to change some aspects related to mindsets around health, although the relationship may depend on the type of remediation as well as larger neighborhood conditions such as racial and income segregation. Such low-cost environmental remediations have the potential to change certain fundamental drivers of poor health in New Orleans and comparable cities. Regardless of whether it is the remediation of vacant and abandoned land and homes in an area or other neighborhood interventions, policymakers should consider methods to minimize potential negative consequences such as gentrification and unequal access. It is important to explore strategies to mitigate displacement, including affordable housing, engaging community members in the approaches taken, and best practices for developing spaces and places that are welcoming to all [34, 35].

Also essential is the attention to the systems and structures within which the spatial patterns of vacant and abandoned properties are shaped. Interventions such as this one are important tests of built environment changes, but such changes must be considered in the wider systems that perpetuate vacancy, abandonment, and other neighborhood disinvestment [36]—the ecosystem of power that has created and maintained inequitable resource distribution [37]. We must continue to attend to the laws, policies, regulations, and budgets that have been drivers of neighborhood conditions, which will take multi-sector coordination and cooperation for change. However, attention to the other structural determinants [37] such as culture, values, and norms as well as governance and institutional practices will be crucial for creating lasting change needed in US neighborhoods and moving the needle on health inequities.

## Data Availability

Data is available at the request of the author.
